# Botulinum toxin type A injection in treatment of pediatric cyclic esotropia

**DOI:** 10.3389/fmed.2026.1911779

**Published:** 2026-08-19

**Authors:** Yida Huang, Tongyao Fu, Xiaoqian Zhang, Xiumiao Li, Chenghu Wang

**Affiliations:** 1 The Affiliated Eye Hospital, Nanjing Medical University, Nanjing, China; 2 The Chinese University of Hong Kong, Shenzhen Hospital, Shenzhen, China; 3 Fourth School of Clinical Medicine, Nanjing Medical University, Nanjing, China

**Keywords:** botulinum toxin type A, cyclic esotropia, nonsurgical treatment, ocular alignment, pediatric strabismus

## Abstract

**Purpose:**

This study aimed to assess the long-term motor, sensory, and safety outcomes following botulinum toxin type A (Botox®) injection for cyclic esotropia in children.

**Methods:**

We retrospectively enrolled children with cyclic esotropia receiving botulinum toxin type A injections between May 2023 and February 2025 who had a minimum 12-month postoperative follow-up. All subjects received complete ophthalmologic assessments comprising best-corrected visual acuity (BCVA), spherical equivalent (SE), and fundus photography, alongside dedicated strabismus evaluations for deviation angle, ocular motility, and binocular function. Ocular deviation and binocular function (distance and near stereopsis) were measured at baseline and scheduled follow-up visits at 1 week, 1, 2, 3, 4, 6 and 12 months postinjection. All adverse events emerging throughout follow-up were prospectively recorded. Treatment success was defined as no recurrence of cyclic esotropia with residual manifest deviation ≤ 5 prism diopters (PD).

**Results:**

Seven pediatric patients (2 males, 5 females; aged 3–5 years) were enrolled in this cohort. At 3 months postinjection, six subjects achieved orthotropia within ± 5 PD, while one developed transient recurrent deviation. Notably, none demonstrated persistent cyclic esotropia. Adverse events were identified in all patients at the 1-week follow-up: upper eyelid ptosis in 6 of 7 patients (85.7%), consecutive exotropia in 6 of 7 (85.7%), and vertical strabismus in 2 of 7 (28.6%). Two new-onset vertical strabismus cases were detected at the 1-month visit, raising the overall prevalence to four patients. Partial recovery was observed in 4/6 (66.7%) ptosis cases, 4/6 (66.7%) consecutive exotropia cases, and none of the four vertical strabismus cases; meanwhile, binocular function improved in 2/7 (28.6%) children. By the 3-month follow-up, complete resolution was seen in all ptosis (6/6, 100%) and consecutive exotropia (6/6, 100%) cases, alongside improvement in 3/4 (75%) vertical strabismus and 6/7 (85.7%) binocular function. At the 12-month endpoint, all seven patients, including the single case with transient early recurrence at 3 months, maintained ocular alignment within ± 5 PD with no recurrent cyclic deviation, and binocular function recovered to near-normal levels across the entire cohort.

**Conclusion:**

Botulinum toxin type A injection effectively interrupts the cyclic deviation pattern and yields durable ocular alignment in this small cohort of children with cyclic esotropia. The majority of injection-related adverse events resolved by 3 months, with complete resolution of all complications by 4 months. Accordingly, botulinum toxin type A injection represents a viable nonsurgical alternative for the management of pediatric cyclic esotropia.

## 1 Introduction

Cyclic esotropia is a rare subtype of strabismus, with an estimated incidence of 1 in 3,000–5,000 individuals (1). It is defined by regularly alternating ocular alignment: manifest esotropia on alternate days interspersed with orthotropia or trivial esodeviation, most frequently following a 48-h cycle; cycles of 72 or 96 h have also been documented (2). This disorder predominantly manifests during childhood, yet its underlying pathogenesis remains incompletely elucidated. Putative pathogenic mechanisms encompass circadian rhythm disorders, fusion insufficiency, and central nervous system regulatory anomalies (3). Given substantial cyclic fluctuations in deviation magnitude, identification of an optimal surgical target angle poses considerable challenges for conventional strabismus surgery. Suboptimal surgical intervention may advance the disease into constant esotropia and irreversible deterioration of binocular function, underscoring the clinical demand for effective nonsurgical alternatives.

Botulinum toxin type A (BTXA; Botox®) causes reversible chemical denervation of extraocular muscles via suppression of acetylcholine release at neuromuscular junctions, which attenuates medial rectus contractility (4, 5). Accumulating evidence supports BTXA injection as an effective intervention for acute comitant esotropia (ACE) (3). Nevertheless, no universally accepted BTXA dosage regimen has been established for strabismus, with published therapeutic doses spanning 2.5–5.0 units tailored to variable ACE deviation severities (6, 7). These encouraging results imply potential utility of BTXA for cyclic esotropia with fluctuating deviation angles. However, the optimal dosing strategy and long-term therapeutic efficacy for this unique strabismus subtype are still undetermined.

This retrospective study enrolled pediatric cyclic esotropia patients treated with BTXA injection to analyze clinical features and therapeutic outcomes. Postinjection ocular alignment, binocular function recovery, and resolution of procedure-related adverse events were assessed to generate clinical evidence guiding nonsurgical management of this rare strabismus.

## 2 Methods

This retrospective clinical study was approved by the Institutional Review Board of the Eye Hospital of Nanjing Medical University and conducted in accordance with the ethical principles outlined in the Declaration of Helsinki. The treatment protocol, anticipated clinical benefits, potential procedural risks and adverse events associated with botulinum toxin type A (BTXA; Botox®, Allergan Pharmaceuticals Ireland) injection were fully explained to all enrolled children and their legal guardians, after which written informed consent was obtained for each participant. Medical charts of patients diagnosed with cyclic esotropia who received BTXA injection between May 2023 and February 2025 at our institution were retrospectively screened. Only subjects completing a minimum 12-month postoperative follow-up were included for final analysis.

### 2.1 Inclusion and exclusion criteria

Inclusion criteria: (1) Met standardized clinical diagnostic criteria for cyclic esotropia; (2) Age ≥3 years at the time of injection; (3) Eligible for BTXA treatment without known drug allergy or procedural contraindications; (4) No concomitant organic ocular disorders except refractive error and primary strabismus; (5) Complete clinical data available for ≥12 months of serial follow-up.

Exclusion criteria: (1) Preexisting amblyopia or other functional ocular abnormalities; (2) Baseline vertical strabismus or combined complex strabismus; (3) Restricted or abnormal extraocular motility; (4) Systemic disease or confirmed central nervous system (CNS) lesions; (5) Prior history of strabismus surgery or previous BTXA injection.

Amblyopia was definitively excluded for all patients according to standardized screening criteria: (1) interocular best-corrected visual acuity difference ≤ 1 line (≤0.1 under decimal recording); (2) no substantial anisometropia, defined as a between-eye spherical equivalent discrepancy ≤1.0 D; (3) unremarkable results on fundoscopy and cycloplegic refraction. Every one of the seven children exhibited bilateral decimal BCVA values between 0.8 and 1.0, matching normative visual thresholds for their age group and confirming the absence of amblyopia.

### 2.2 General ophthalmic assessment

All participants underwent full ophthalmic workup, including uncorrected visual acuity, best-corrected visual acuity (BCVA), cycloplegic refraction and spherical equivalent (SE) calculation plus dominant-eye identification. Anterior segment evaluation was completed via slit-lamp biomicroscopy, and standardized fundus photography (Canon Inc., Japan) was acquired for every patient.

### 2.3 Deviation angle measurement

Prism alternate cover testing under full refractive correction was applied to quantify manifest deviation; interphase deviation differences between esotropic and orthotropic cycles were documented individually.

### 2.4 Ocular motility assessment

Monocular duction testing was performed by occluding one eye while the fellow eye tracked a fixation target across all cardinal gaze directions to identify restrictive or paretic motility defects. Binocular versions were examined across nine diagnostic gaze positions to grade underaction or overaction of extraocular muscles.

### 2.5 Binocular function testing

Synoptophore examination (66 Vision Tech, Suzhou, China) was used to grade simultaneous perception (Grade I), fusional ability (Grade II), and distance stereopsis (Grade III). Near stereopsis was quantified using the Titmus stereo test (Stereo Optical, USA), graded as macular stereopsis (≤200 arcsec), peripheral stereopsis (400–800 arcsec), or absent stereopsis (>800 arcsec). Given limited cooperation in young pediatric subjects, only Grade III distance stereopsis and Titmus near stereopsis data were included for final statistical analysis.

### 2.6 Cyclic feature recording

The duration of manifest esotropia and the intermittent interval of orthotropic phases were precisely recorded to characterize individual cyclic patterns.

### 2.7 Preoperative neurological screening

All seven patients underwent formal neurological consultation prior to BTXA administration, consisting of thorough clinical history review and complete neurological physical examination. Per the attending neurologist’s clinical judgment, routine brain MRI was withheld for patients presenting no neurological signs or symptoms. Although brain magnetic resonance imaging was only conducted in a subset of participants, all scans ruled out Chiari type I malformation and other major structural central nervous system abnormalities. We acknowledge that this selective neuroimaging strategy constitutes a limitation of our preoperative screening workflow.

### 2.8 Injection protocol and surgical technique

Anesthesia modality (general or topical anesthesia) was selected based on patient age and clinical cooperation. Commercially available lyophilized BTXA (50 U per vial) was reconstituted with 1 mL sterile 0.9% normal saline immediately before injection. Following adequate anesthesia, a Parks inferonasal conjunctival peritomy was created bilaterally. Under operating microscope visualization, the medial rectus muscle was isolated using a strabismus hook, and 0.1 mL (5 U) BTXA solution was injected into the muscle belly 10 mm posterior to the medial rectus insertion site. Gentle local compression was applied at the injection site for 1 min, and conjunctiva was closed with a single 8-0 coated Vicryl absorbable suture (Polyglactin 910, Ethicon, USA). All seven patients received bilateral 5-U BTXA injections, with all procedures performed by a single experienced strabismus surgeon to minimize interoperator variability. A uniform dose of 5-U per medial rectus was selected based on published pediatric dosing for acute comitant esotropia, the inherent fluctuation of deviation in cyclic esotropia, and the practical advantages of single-session bilateral injection in young children.

### 2.9 Outcome definitions and follow-up assessments

Serial assessments of deviation magnitude and binocular function were scheduled at baseline, 1 week, and 1, 2, 3, 4, 6 and 12 months postinjection. Notably, this retrospective study lacked scheduled follow-up aligned with patients’ cyclic phases, so deviation measurements were not standardized to individual disease cycles. All procedure-related adverse events, their onset timelines, and timings of spontaneous improvement or complete resolution were prospectively documented throughout follow-up. Treatment success was predefined as permanent elimination of cyclic esotropia with residual primary-position manifest deviation ≤ 5 prism diopters (PD).

### 2.10 Statistical analysis

Statistical analyses were conducted using SPSS 23.0 software (IBM, USA). Owing to the small sample size and non-normal data distribution, nonparametric statistical methods were adopted. Continuous variables were presented as median with interquartile range (IQR). Longitudinal changes across sequential follow-up time points were compared via the Friedman test. Pairwise comparisons against baseline were performed using the Wilcoxon signed-rank test with Holm’s sequential Bonferroni correction for multiple testing. A two-tailed *p* value < 0.05 was defined as statistically significant.

## 3 Results

### 3.1 Clinical and demographic characteristics of the patients

This study enrolled seven pediatric patients, including 2 males and 5 females. The median age at BTXA injection was 3.0 years (interquartile range [IQR], 3.0–5.0 years; full range, 3–5 years). The median pretreatment disease duration was 7 months (IQR, 2.5–9.5 months). Regarding cyclic strabismus features, the median duration of each esotropic episode was 17 h (IQR, 9.25–24 h), and the median interval between alternating episodes was 48 h (IQR, 24–48 h) (Table 1). A Mann–Whitney *U* test was performed to compare baseline ocular parameters between the right and left eyes. No significant interocular differences were detected in spherical equivalent (SE) or best-corrected visual acuity (BCVA). The median SE values were 1.75 D (IQR, 0.13–1.88 D) for the right eyes and 1.75 D (IQR, 0.19–1.94 D) for the left eyes (*U* = 22.0, two-tailed *p* = 0.7961). The median BCVA (decimal) was 1.0 (IQR, 0.93–1.0) in the right eyes and 1.0 (IQR, 0.85–1.0) in the left eyes (*U* = 25.5, two-tailed *p* = 0.936). These baseline data confirmed symmetrical refractive status and balanced visual acuity bilaterally in all enrolled patients.

**Table 1:** Clinical and demographic characteristics of the 7 patients.

Characteristic	Patient (*n* = 7)		*p* value^*^
Age at surgery (years)			
M (Q1, Q3)	3 (3, 5)		–
Sex			
Male	2		–
Female	5		–
Duration (months)			
M (Q1, Q3)	7 (2.5, 9.5)		–
Spherical equivalent (SE, diopters)	OD	OS	
M (Q1, Q3)	1.75 (0.13, 1.88)	1.75 (0.19, 1.94)	0.7961^#^
Best-corrected visual acuity (BCVA)	OD	OS	
M (Q1, Q3)	1.0 (0.93, 1.0)	1.0 (0.85, 1.0)	0.936^#^
Attack duration (hours)			
M (Q1, Q3)	17 (9.25, 24)		–
Attack interval (hours)			
M (Q1, Q3)	48 (24, 48)		–

Comparisons between groups were performed using the ^#^Mann–Whitney *U* test. ^*^p < 0.05 indicated statistical significance. SE, spherical equivalent; BCVA, best-corrected visual acuity measured with the Standard Logarithmic Visual Acuity Chart (Chinese National Standard GB 11533-2011) and documented in decimal notation.

### 3.2 Effect of BTXA injection on ocular alignment

Of the seven enrolled patients, six achieved satisfactory ocular alignment within 3 months post-injection, with 85.7% (6/7) presenting a deviation of ± 5 prism diopters (PD) in the primary gaze position. One patient had a transient recurrence of symptoms at the 3-month follow-up; nevertheless, this cyclic manifestation completely resolved by the 12-month assessment. Ultimately, all patients maintained a deviation within ± 5 PD in the primary position throughout the long-term follow-up.

The median deviation angles (interquartile range [IQR], PD) were +60 (+45 to +60) at baseline, −50 (−50 to −35) at 1 week, −15 (−47.5 to −15) at 1 month, −5 (−15.5 to +3) at 2 months, −2 (−2.5 to +2.5) at 3 months, and +3 (0 to +4) at 4 months (Table 2). A Friedman test revealed a statistically significant temporal change in deviation (*χ*^2^ [5] = 25.95, *p* < 0.001; Kendall’s W = 0.74). Post-hoc pairwise comparisons against baseline were performed using the Wilcoxon signed-rank test, which identified significant reductions in deviation at all follow-up time points: 1 week (median change, −90.0 PD; 95% confidence interval (8), −115.0 to −62.5), 1 month (−78.0 PD; 95% CI, −105.0 to −51.0), 2 months (−56.3 PD; 95% CI, −85.0 to −35.0), 3 months (−53.5 PD; 95% CI, −63.0 to −28.5), 4 months (−52.5 PD; 95% CI, −60.0 to −30.0), 6 months (−53.3 PD; 95% CI, −60.0 to −44.0), and 12 months (−53.0 PD; 95% CI, −60.0 to −46.0). All Holm-adjusted *p* values were less than 0.05.

**Table 2:** Medians [IQR], paired median changes vs. baseline, and orthotropia rates across follow-ups.

Time point	Angle of deviation, median [IQR], PD	Within ± 5 PD, *n*/*N* (%)	HL Δ vs. baseline (95% CI), PD	*p* (Δ vs. baseline)
Baseline (preoperative attack duration)	60.0 [45.0, 60.0]	0	–	–
After 1 week	−50.0 [−50.0, −35.0]	1/7 (14.3%)	−90 (−115.0 to −62.5)	*
After 1 month	−15.0 [−47.5, −15.0]	1/7 (14.3%)	−78 (−105.0 to −51.0)	*
After 2 months	−5.0 [−15.5, +3.0]	2/7 (28.6%)	−56.3 (−85.0 to −35.0)	*
After 3 months	−2.0 [−2.5, +2.5]	6/7 (85.7%)	−53.5 (−63.0 to −28.5)	*
After 4 months	+3.0 [0, +4.0]	6/7 (85.7%)	−52.5 (−60.0 to −30.0)	*
After 6 months	0 [−0.5, +1.5]	7/7 (100%)	−53.5 (−60.0 to −44.0)	*
After 12 months	0 [0, 0]	7/7 (100%)	−53.0 (−60.0 to −46.0)	*

HL Δ vs. Baseline: Hodges-Lehmann estimate of paired differences (follow-up value minus baseline value for deviation angle), with 95% confidence intervals (CI). *p* values were calculated using the Wilcoxon signed-rank test versus baseline, with the Holm method applied to adjust for seven multiple comparisons. Significance level: ^*^p < 0.05. PD, prism diopters; IQR, interquartile range.

**Figure 1:**
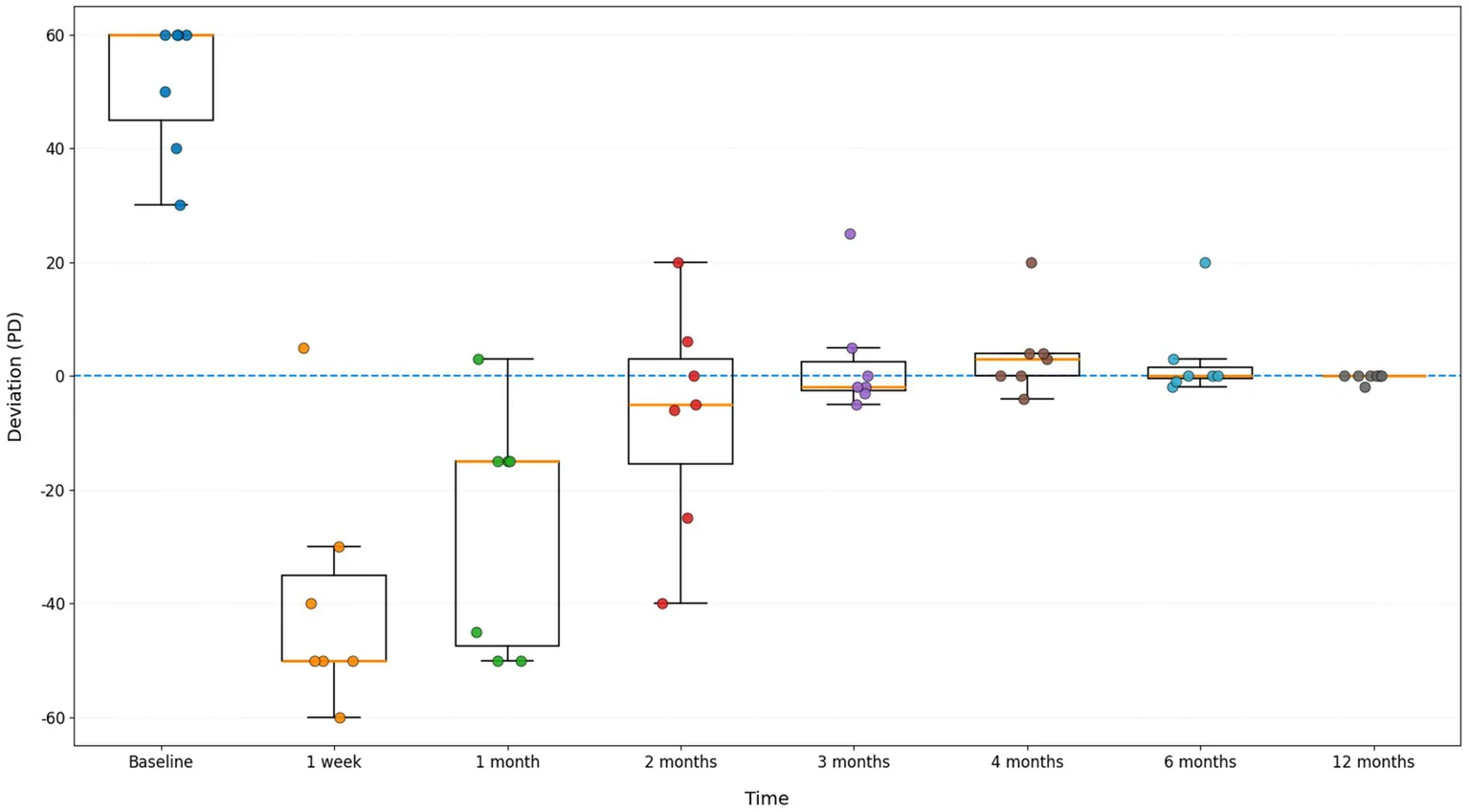
Longitudinal changes in deviation angle following BTXA injection. Box plots illustrate the median (yellow solid line) and interquartile range (IQR) at each follow-up time point, with overlaid individual patient data (*n* = 7). The blue dashed line represents orthotropia (0 PD). Baseline measurements were acquired during the preoperative esotropic phase.

From the 3-month time point onwards, 85.7% (6/7) of patients sustained stable ocular alignment within ± 5 PD. Box plots overlaid with individual data points (Figure 1) visually demonstrate the dynamic alterations in deviation angles across all follow-up visits.

### 3.3 Recovery of binocular function after BTXA treatment

At baseline preoperative esotropic phases, all patients exhibited absent grade III distance stereopsis (synoptophore) and negative Titmus near stereopsis. During the preoperative orthotropic interphase, only one patient presented measurable distance stereopsis, while two patients demonstrated detectable near stereopsis (40 arcsec and 50 arcsec). At 2, 3, and 4 months postinjection, the number of patients who recovered grade III distance stereopsis increased sequentially to 3, 5, and 6, respectively. Correspondingly, Titmus near stereopsis (median [IQR], arcsec) improved progressively to 100 [80, 140], 90 [57.5, 100], and 50 [50, 60] at the identical time points. At the 3-month follow-up, 85.7% (6/7) of patients showed measurable improvement in binocular function. By the 12-month final assessment, all seven patients achieved nearly normal binocular function.

### 3.4 Postoperative complications and overall treatment course

**Figure 2:**
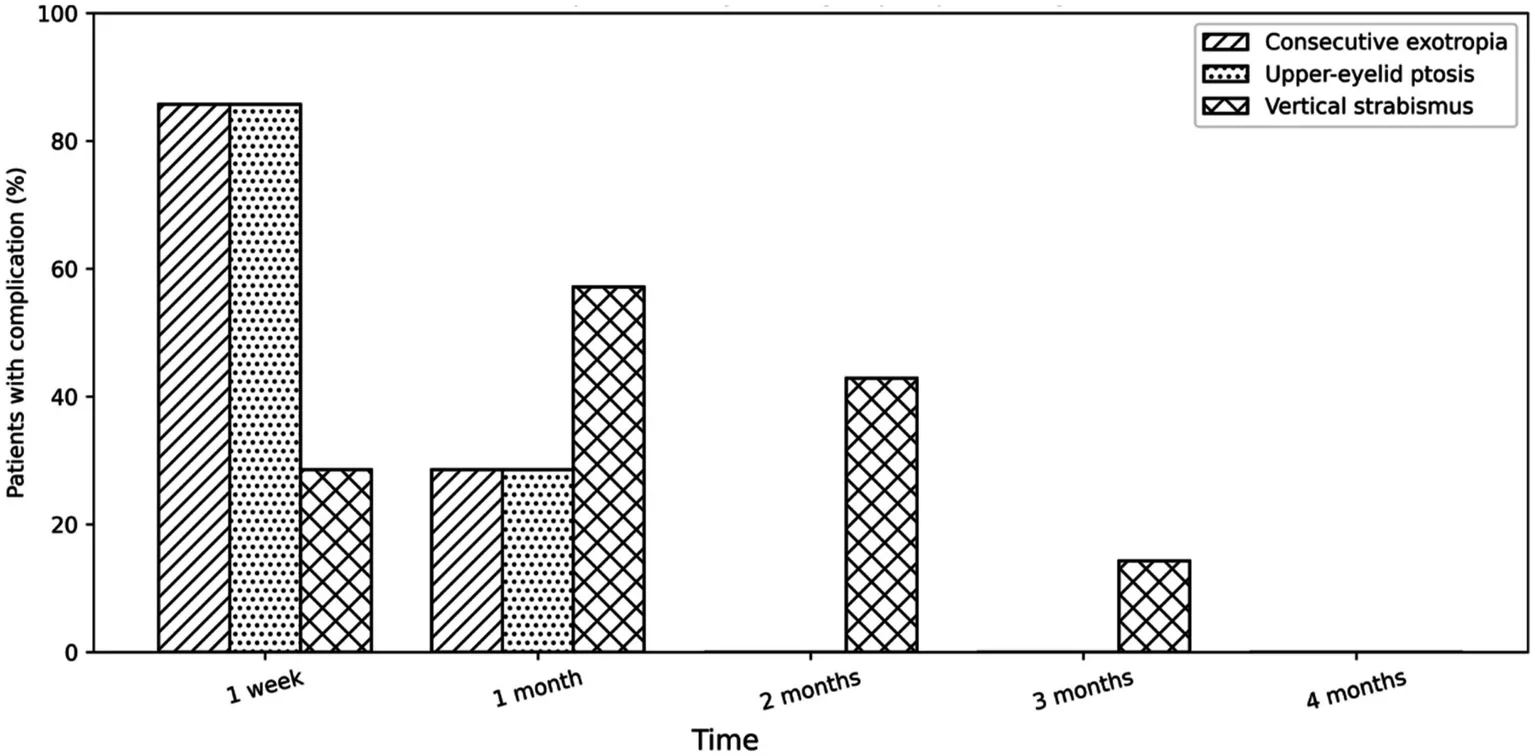
Temporal prevalence of postoperative complications following BTXA injection. Bars represent the percentage of patients with consecutive exotropia, upper-eyelid ptosis, or vertical strabismus at each follow-up visit. Percentages were calculated using the total cohort as the denominator (*n* = 7), and individual patients could contribute to more than one complication category.

All seven patients developed at least one complication at the 1-week postinjection follow-up. Specifically, consecutive exotropia and upper eyelid ptosis each occurred in 85.7% (6/7) of patients, while vertical strabismus was observed in 28.6% (2/7) of patients. Two new-onset vertical strabismus cases were identified at the 1-month visit, raising the overall prevalence of vertical deviation to 57.1% (4/7). Compared with the 1-week postoperative status, 66.7% (4/6) of consecutive exotropia cases and 66.7% (4/6) of ptosis cases showed partial improvement at 1 month, whereas no improvement was observed in vertical strabismus. By 2 months, complete resolution was achieved for all cases of consecutive exotropia (6/6, 100%) and ptosis (4/4, 100%), while only 25% (1/4) of vertical strabismus cases improved. The resolution rate of vertical strabismus increased to 75% (3/4) at 3 months. Ultimately, all procedure-related adverse events resolved completely within 4 months after BTXA injection. The dynamic changes in complication prevalence across follow-up time points are illustrated in Figure 2. The figure depicts the longitudinal trends of consecutive exotropia, upper eyelid ptosis, and vertical strabismus in the entire cohort (*n* = 7). Notably, these three adverse events were not mutually exclusive. The graphical data further demonstrate rapid and complete resolution of exotropia and ptosis by 2 months, whereas vertical strabismus resolved more gradually, with full resolution achieved by 4 months postoperatively.

**Figure 3:**
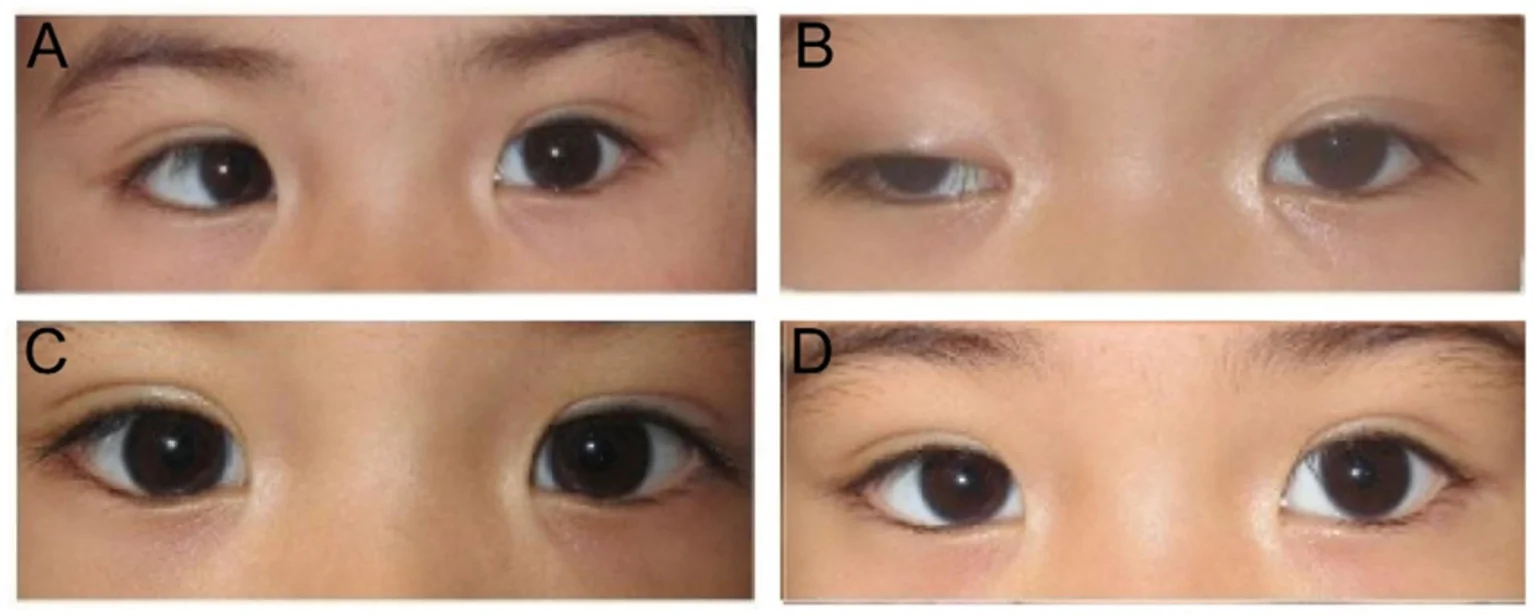
Representative primary-position corneal light reflex photographs of one typical pediatric patient with cyclic esotropia before and after BTXA injection. **(A)** Pre-injection esotropic phase (baseline esotropia); **(B)** 1 week post-injection (consecutive exotropia and upper eyelid ptosis); **(C)** 6 months post-injection (orthotropia); **(D)** 12 months final follow-up (orthotropia).

To intuitively demonstrate the dynamic changes of ocular alignment throughout follow-up after BTXA injection, serial primary-position photographs from one representative enrolled child are presented in Figure 3, which illustrates the characteristic cyclic manifestation pre-treatment and gradual alignment normalization during long-term follow-up.

## 4 Discussion

Cyclic esotropia is a rare, distinct strabismus subtype with incompletely clarified pathogenesis. Its pathophysiology is largely ascribed to impaired neural control of binocular fusion, possibly stemming from subtle circadian rhythm disruption or altered neuromodulatory signaling. The circadian etiology hypothesis remains unvalidated (3, 9). Indirect supporting evidence includes a pediatric case in which cyclic strabismus vanished abruptly following rapid transmeridian travel across multiple time zones (3, 10). The classic 48-h alternating cycle features pronounced esotropia on 1 day and orthotropia or trivial esodeviation on the alternate day, while atypical 24-h or 72-h periodic patterns have also been documented (11, 12). This disease predominantly arises in preschool children aged 3–4 years with acute onset, occasionally triggered by febrile illness, fright, or head trauma. On esotropic days, patients present with large-angle inward deviation, lost single binocular vision, diplopia and compromised stereopsis. Orthotropic days are associated with full ocular alignment and restored binocularity in some patients, whereas others retain mild residual esotropia. Without timely intervention, a substantial proportion progresses to constant esotropia over months to years, accompanied by irreversible binocular function decline and elevated amblyopia risk in visually immature children, highlighting the clinical need for early effective management.

Extraocular muscle surgery constitutes conventional definitive treatment, with mainstream procedures including bilateral medial rectus recession or unilateral medial rectus recession combined with lateral rectus resection. Surgical targets are commonly set based on esotropic-day deviation magnitude to prevent spontaneous disease fixation toward constant strabismus, yet late-onset recurrent or consecutive strabismus remains a frequent postoperative complication (3, 11, 13, 14). Though Souza-Dias et al. documented favorable long-term surgical efficacy in pediatric cohorts (15), operative intervention carries inherent risks such as scleral perforation, slipped extraocular muscles, and over- or undercorrection under general anesthesia, alongside procedural discomfort under topical anesthesia.

Multiple nonsurgical alternatives have therefore been explored. Systemic baclofen and topical cholinesterase inhibitors (diisopropyl fluorophosphate, echothiophate iodide) and miotic agents yielded unsatisfactory therapeutic effects across prior clinical trials (3). Optical prism management represents a noninvasive alternative with inconsistent therapeutic outcomes (16). Voide et al. reported permanent cyclic pattern interruption and sustained 24-month remission in one single case after prism correction, postulating cyclic esotropia originates from decompensated latent deviation and recommending prisms as first-line therapy with surgery reserved for refractory cases (17). Conversely, Wright et al. demonstrated substantial vision deterioration and fusion breakdown related to high-power Fresnel prisms: 20-PD Fresnel prisms reduced visual acuity by four lines versus two lines with ground-in prisms, and 25-PD Fresnel lenses disrupted fusion in 80% of treated eyes compared with 20% for conventional solid prisms (16). Wipf et al. reported three children receiving single bilateral 5-U BTXA injections with follow-up ranging from 8 to 16 months (18). Their study only documented final alignment outcomes, without serial deviation measurements, sequential stereopsis recovery data, or systematic monitoring of injection-related adverse events. Multiple small case series have confirmed sustained BTXA efficacy over 4–8 years of follow-up, yet isolated late recurrence at 16 months has been previously documented, highlighting the necessity of long-term postoperative surveillance (19). Our cohort of seven patients represents the largest single-center pediatric series published to date, with all participants managed under a standardized one-time bilateral 5-U BTXA protocol. We conducted structured serial assessments at predefined timepoints: 1 week, 1, 2, 3, 4, 6 and 12 months, and adopted a clear, reproducible therapeutic endpoint: complete resolution of cyclic strabismus with residual deviation ≤ 5 PD. We systematically quantified progressive recovery of distance and near stereopsis across all visits and mapped the full resolution timeline of ptosis, consecutive exotropia and vertical strabismus, verifying that all adverse events resolved spontaneously within 4 months. These detailed longitudinal profiles of safety and sensory restoration have not been comprehensively described in prior reports. Collectively, our standardized serial longitudinal data, unified dosing regimen, systematic safety characterization, and novel observation of transient early deviation flare substantially expand the limited body of evidence regarding BTXA therapy for pediatric cyclic esotropia.

Produced by *Clostridium botulinum*, BTXA induces reversible chemical denervation and transient extraocular muscle paresis lasting roughly 8–12 weeks after intramuscular injection. Temporary weakness of the injected medial rectus shifts oculomotor balance via relative overaction of antagonistic lateral rectus muscles. Transient postoperative over- or under- correction facilitates extraocular muscle remodeling and central sensory-motor readjustment, thereby re-establishing physiological fusion and sustaining long-term orthotropia (20). However, this remains merely a hypothesis, as the current study did not acquire longitudinal objective data to offer direct evidence validating this mechanism. Numerous potential mechanisms have been put forward to explain why BTXA yields lasting therapeutic benefits long after its peripheral paralytic pharmacologic effect fades. Croes et al. (21) proposed that botulinum neurotoxin could produce persistent corrective effects on immature extraocular muscles, driven by central nervous system adaptive plasticity rather than irreversible peripheral muscle changes. Ugalde et al. (22) observed robust myofiber remodeling and satellite cell activation in rabbit extraocular musculature post BTXA injection, while Kang et al. (23) documented durable long-term alignment outcomes after BTXA treatment for acquired non-accommodative esotropia. Collectively, these works indicate sustained BTXA efficacy arises from multifactorial pathways, encompassing central sensory-motor recalibration, morphological restructuring of extraocular muscles, and the recovery of stable fusional function. Even so, the exact causal cascades linking these processes to lasting strabismus correction have not been fully characterized. Against this mechanistic framework, our clinical observations align with the theory that transient chemical denervation opens a critical window to facilitate binocular sensory readjustment. That said, our study was not designed to verify this theoretical model empirically. Further dedicated research is therefore necessary to unpack the distinct signaling and neural pathways responsible for the enduring therapeutic response seen in children with cyclic esotropia.

Distinct from incisional strabismus surgery, BTXA avoids permanent anatomical alteration of muscle insertion and conjunctival scarring, featuring favorable repeatability and full pharmacological reversibility. Despite short-lived myoparalytic action, therapeutic benefits frequently persist long after toxin clearance. BTXA has gained widespread clinical acceptance for acute comitant esotropia (ACE) and adult cyclic esotropia, with accumulating evidence supporting its application in pediatric populations (8, 24). Potential adverse effects include ptosis, overcorrection, and vertical strabismus. Nevertheless, high-quality head-to-head comparative data remain scarce given cyclic esotropia’s low incidence and heterogeneous clinical manifestations.

Consensus regarding optimal intervention timing for cyclic esotropia is lacking. Several clinicians propose watchful waiting while partial binocularity persists on orthotropic days, reserving surgery only after progression to constant deviation (20). Based on our cumulative clinical experience with both surgical and injectable management, we advocate early BTXA intervention to avoid permanent binocular damage secondary to chronic fixed esotropia. In our cohort, ocular alignment improved rapidly post-injection: 6/7 subjects achieved alignment within ± 5 PD by 3 months, and all seven maintained satisfactory positioning at the 12-month endpoint. Binocular function recovery initiated as early as 2 months posttreatment, with over half recovering complete distance and near stereopsis by month 4, consistent with published clinical findings (6, 25). All injection-related adverse events (ptosis, consecutive exotropia, vertical strabismus) were self-limited and fully resolved within 4 months, verifying BTXA’s favorable safety profile as a nonsurgical regimen.

One participant presented transient esotropic rebound at the 3-month follow-up. This child demonstrated 30 PD manifest deviation on esotropic days plus 10–15 PD residual esodeviation during intermission at baseline, with complete absence of both distance and near stereopsis. At 3 months postinjection, recurrent +25 PD esotropia emerged without restoration of cyclic periodicity. Distance stereopsis remained absent, and near stereopsis measured 400 arcsec. Deviation gradually decreased to +20 PD alongside improved near stereopsis (200 arcsec) by month 4. Serial telephone follow-up confirmed continuous monthly improvement without cyclic relapse, and the patient achieved full orthotropia with measurable distance stereopsis and 60 arcsec near stereopsis at the 12-month visit. We attribute this temporary deviation flare to physiological sensory adaptation overlapping waning BTXA chemodenervation rather than definitive treatment failure. Since BTXA’s paralytic effect subsides within 8–12 weeks, residual latent deviation may become transiently unmasked before central fusion stabilization. For patients presenting with mild recurrent deviation, progressive stereopsis improvement and absent cyclic recurrence, close periodic monitoring instead of immediate repeat injection prevents unnecessary overtreatment. Recurrent cyclic esotropia and consecutive exotropia are well-documented postoperative complications after BTXA surgery (6, 25). While large-scale controlled comparative trials remain absent, our longitudinal outcomes validate BTXA’s capacity to abolish cyclic patterns and support its role as a reasonable first-line therapy. The rationale for delivering a standardized bilateral 5 U dose to each medial rectus muscle rested on three key clinical considerations: (a) No validated dose–strabismus angle nomogram has been established specifically for cyclic esotropia. We therefore adopted dosing ranges reported for BTXA in pediatric acute comitant esotropia (2.5–5.0 U), combined with our institutional clinical experience managing childhood strabismus. (b) Deviation angles in cyclic esotropia exhibit marked periodic variability. Measurements captured during esotropic manifest phases tend to overestimate the baseline oculomotor imbalance, while readings taken during orthotropic intermissions underestimate it. Dose stratification based on a single time-point measurement is thus inherently unreliable. (c) The cohort’s median age was only 3 years old. A single bilateral injection avoids the repeated hospital visits and repeated injections required for staged or titrated dosing protocols, substantially reducing procedural distress and psychological burden for young pediatric patients. We recognize that this one-size-fits-all dosing regimen may partly explain the variability in early therapeutic responses. Theoretically, patients presenting with larger baseline deviations could attain superior alignment with escalated doses or refined injection placement. Notably, the single case with transient deviation rebound at month 3 cannot be attributed to insufficient dosing: this child’s cyclic deviation spanned 30 PD during manifest episodes and 10–15 PD during intermissions, corresponding to a moderate angle rather than the most severe within our series. Even so, the fixed-dose design prevents robust characterization of dose–response associations. Encouragingly, all seven participants, including the patient with temporary early recurrence, maintained stable ocular alignment within ± 5 prism diopters at the 12-month primary endpoint. This confirms that the uniform 5 U dose yielded satisfactory long-term efficacy across the full spectrum of deviation severities observed in our cohort. We have explicitly listed our limited sample size as a core study limitation, as it precluded subgroup analyses to quantitatively examine dose-effect relationships. Future prospective work should develop stratified dosing frameworks incorporating baseline deviation magnitude, extraocular muscle anatomical features, and patient age to refine personalized BTXA treatment protocols for pediatric cyclic esotropia.

Beyond these considerations, several methodological limitations specific to the study of cyclic esotropia warrant discussion. Baseline variability in preoperative deviation magnitude and stereopsis function further restricts external validity of our results. Follow-up assessments were not prospectively scheduled to align with each patient’s unique cyclic strabismus phase. In the early post-injection interval prior to full resolution of cyclic alternation, measured deviation angles at each clinic visit could be confounded by whichever cyclic state the patient presented in at the time of examination. Nevertheless, multiple key findings from our cohort help alleviate this methodological concern: (a) Cyclic strabismus alternation was fully resolved in all patients by 3 months post-injection, greatly minimizing cycle-related interference with primary outcome measurements collected at the 3-, 4-, 6-, and 12-month follow-up timepoints; (b) Every patient exhibited a uniform longitudinal trend of reduced deviation from baseline across serial visits, verifying that any residual cyclic fluctuations did not mask the core therapeutic effect of BTXA injection; (c) All seven subjects met our prespecified treatment success endpoint at the 12-month assessment, defined as sustained ocular alignment within ± 5 prism diopters with no return of cyclic esotropia, which confirms consistent, long-lasting therapeutic efficacy overall.

Future multicenter investigations with larger stratified cohorts are required to clarify BTXA dose-effect correlations by baseline deviation severity, cyclic periodicity and patient age. Prospective protocols could identify predictive biomarkers of sustained remission, such as early stereopsis recovery within 1–2 months, and refine personalized injection timing relative to individual cyclic phases to optimize dosage criteria for initial treatment versus delayed re-injection. Additional variables including single esotropic episode duration and injection timing across alternating cycles require further exploration to standardize BTXA therapeutic algorithms for pediatric cyclic esotropia. Several limitations of this study should be acknowledged. First, its retrospective, single-center, uncontrolled design precludes randomization and parallel control comparisons. Accordingly, our results are vulnerable to temporal trends, observer bias, and unmeasured confounding factors, including the natural clinical course of cyclic esotropia. Cyclic esotropia may undergo spontaneous resolution or progress to constant esotropia, further weakening the strength of causal inference. Second, the small sample size (*n* = 7) leads to insufficient statistical power, making any subgroup analyses impractical. Third, the predominance of female patients (5/7) restricts the generalizability of our findings across sexes. Nevertheless, consistent gender predilection has not been documented in previous literature, implying that this skewed sex ratio most likely arises from sampling variation. Although standardized examination protocols and unified treatment regimens were adopted to mitigate these biases, large-scale, multicenter, prospective randomized controlled trials are still required to validate our observations.

## Data Availability

The original contributions presented in the study are included in the article/supplementary material, further inquiries can be directed to the corresponding author/s.
